# Accuracy of heparin-binding protein for the diagnosis of nosocomial meningitis and ventriculitis

**DOI:** 10.1186/s13054-022-03929-x

**Published:** 2022-03-08

**Authors:** Yueyue Kong, Yi Ye, Jiawei Ma, Guangzhi Shi

**Affiliations:** Department of Critical Care Medicine, Beijing Tiantan Hospital, Capital Medical University, Beijing, China

**Keywords:** Cerebrospinal fluid, Heparin-binding protein, Procalcitonin, Lactate

## Abstract

**Background:**

The sensitive and accurate diagnosis of nosocomial meningitis and ventriculitis is still a critical problem. This study was designed to explore the diagnostic value of cerebrospinal fluid heparin-binding protein (HBP) in nosocomial meningitis and ventriculitis in comparison with procalcitonin and lactate.

**Methods:**

In this observational study, 323 suspected patients were enrolled, of which 42 participants were excluded because they could not be accurately grouped, 131 subjects who were eventually diagnosed with nosocomial meningitis or ventriculitis and 150 patients in whom infection was ultimately ruled out were included in the final analysis. The main results are expressed as medians (interquartile ranges). The Chi-squared test was used to compare the baseline characteristics. The Mann–Whitney U-test was used for group and subgroup analyses. The area under the receiver operating characteristic curve was calculated to describe the diagnostic accuracy of the biomarkers. Spearman's partial correlation was used to analyze associations between the biomarkers. Statistical significance was set when *p* value < 0.05.

**Results:**

HBP achieved the largest area under the receiver operating characteristic curve, which was 0.99 (95% confidence interval 0.98—1.00) compared with 0.98 (95% confidence interval 0.96—0.99) for lactate and 0.69 (95% confidence interval 0.62—0.75) for procalcitonin. With a cutoff level at 23 ng/mL, HBP achieved a sensitivity of 97%, a specificity of 95%, a positive predictive value of 93% and a negative predictive value of 98%. The levels of HBP presented no significant discrepancy between patients who received previous empiric anti-infective therapy and those who did not (*p* > 0.05). Higher concentrations of HBP were present in patients with positive microbiological findings (*p* < 0.05). Levels of HBP positively correlated with polymorphonuclear cell count (Spearman's rho = 0.68, *p* < 0.01), white blood cell count (Spearman's rho = 0.57, *p* < 0.01) and lactate (Spearman's rho = 0.34, *p* < 0.01).

**Conclusions:**

Cerebrospinal fluid heparin-binding protein is a reliable auxiliary diagnostic marker that is preferable over lactate and procalcitonin in identifying nosocomial meningitis and ventriculitis, and it also contributes to solving the diagnostic difficulties caused by empiric antibiotherapy.

## Introduction

Nosocomial meningitis and ventriculitis in adults mainly results from invasive neurosurgical procedures or head trauma [1]. It remains a life-threatening complication contributing to considerable morbidity [2, 3]. Patients with a drainage device may have an even higher incidence [4–6]. The syndrome can lead to severe sequelae and increased mortality [7, 8] and poses a particularly great threat to elderly people [9]. Given the adverse clinical outcomes [10, 11], empiric antibiotic therapy is usually initiated in the absence of bacteriologic findings. Cerebrospinal fluid (CSF) cytochemical parameters, including cell count, glucose, protein and lactate suffer from a lack of specificity [12, 13]. The lack of widely recognized cutoff values for these markers makes their interpretation difficult. Sensitivity for Gram staining is unacceptably low [7, 12, 13]. Culture, the gold standard, has an inherent delay of several days to obtain results. A sensitive and reliable biomarker is therefore required.

Heparin-binding protein (HBP) is rapidly mobilized and released from activated neutrophils in response to infections in the early stage of inflammation [14, 15], which makes it a promising biomarker with emerging roles in the diagnosis of infectious diseases. The protein has demonstrated its superiority over procalcitonin (PCT) and C-reactive protein in early recognition of infections [16, 17]. Similarly, HBP showed more powerful diagnostic capability in distinguishing bacterial meningitis from viral meningitis than CSF cytochemical markers [18–20]. However, published results regarding the accuracy of HBP in diagnosing postoperative meningitis and ventriculitis are lacking.

The present study was conducted to assess the value of CSF HBP in identifying meningitis and ventriculitis in patients following neurosurgery compared with CSF lactate and procalcitonin.

## Methods

### Study design and patient population

This single-center observational study was conducted at Beijing Tiantan Hospital, Capital Medical University, between August 2020 and June 2021 and was approved by the Ethics Committee of the hospital. The hospital is the China National Clinical Research Center for Neurological Diseases and most of the neurosurgical patients admitted are patients with central nervous system tumors. Eligible subjects were patients who were older than 18 years and experienced neurosurgery that involved dura opening. The exclusion criteria were community-acquired meningitis, meningitis or ventriculitis diagnosed in other centers before admission, infections secondary to spinal surgery or surgery that only involved blurring holes or shunts. Patients who received empiric antibiotic therapy before sampling were not excluded. The implementation of glucocorticoids and antimicrobial prophylaxis was integrated into routine treatment in patients following neurosurgery in our center. Patients who stayed in the intensive care unit (ICU) for more than 48 h were defined as critically ill.

### Diagnostic criteria

We adopted an adjusted CDC/NHSN definition and specified the cutoffs of white blood cell (WBC) count, protein and glucose in CSF for transparency and reproducibility [21]. Nosocomial meningitis or ventriculitis was confirmed when a patient met criteria 1 or criteria 2 of the definition: 1. Positive cultures of CSF; 2. At least one of these clinical symptoms cannot otherwise be explained by other causes (body temperature > 38 ℃ or headache or meningeal signs or focal neurological impairments) and at least one of the following: 1) white blood cell count > 100 cells/mm^3^, protein > 50 mg/dL and glucose < 2.5 mmol/L in CSF; 2) positive findings on Gram staining of CSF; 3) positive cultures of blood. External ventricular drainage (EVD)-related infection was defined according to Clinical Practice Guidelines for Healthcare-Associated Ventriculitis and Meningitis from Infectious Diseases Society of America (IDSA)[22].

### Data collection

Cerebrospinal fluid was sampled for glucose, protein, cell count, culture, HBP, PCT and lactate analysis in patients suspected to have central nervous system infections following neurosurgery. Patients were enrolled as the infected group when a diagnosis of meningitis or ventriculitis was established according to the criteria listed above. Patients with white blood cells ≤ 100 cells/mm^3^ and glucose ≥ 2.5 mmol/L were enrolled as the control group. Patients who could not be clearly classified were not included in the final statistical analysis. Data involving demographic characteristics, preexisting medical conditions, surgery descriptions and clinical manifestations were collected.

CSF samples for the determination of HBP and PCT were centrifuged and analyzed using Fluorescent Immunalyzer Model AFS-1000 (Guangzhou Labmis Biotech Co. Ltd, Guangzhou, China) according to the manufacturer's instructions. The analytical sensitivity was 0.05 ng/mL for PCT and 0.01 ng/mL for HBP. CSF samples for cytochemical examination (including CSF lactate) were tested in our center's clinical chemistry laboratories.

### Statistical analysis

Given the nonnormality, the main results were expressed as medians (interquartile ranges [IQR]). The Chi-squared test was used to compare the baseline characteristics. The Mann–Whitney U-test was used for group comparisons and subgroup analyses. The area under the receiver operating characteristic curve (AUC) was calculated to describe the diagnostic accuracy of CSF biomarkers. Since previous studies showed positive correlations among WBC, HBP and lactate[19, 20], Spearman's partial correlation was used to analyze the correlations between the biomarkers by controlling the effect of one biomarker and analyzing the correlation between the other two. Statistical significance was set when *p* value < 0.05. All statistics were analyzed using GraphPad Prism version 8.0 (GraphPad Software, USA) and SPSS version 25.0 (IBM Corporation, USA).

## Results

### Baseline characteristics

Cerebrospinal fluid was sampled from 323 suspected patients, and 42 were eventually excluded because of the inability to determine the presence or absence of an infection. Analysis was thereby performed on 281 patients, including 131 patients who were diagnosed with meningitis or ventriculitis as the infected group and 150 patients in whom infection was eventually ruled out as the control group. A total of 325 samples were analyzed, including 131 samples from the infected group and 194 samples from the control group.

The baseline characteristics of the two groups are exhaustively described in Table 1. There were no statistical differences in data involving demographic characteristics and previously existing medical conditions. Among all the characteristics related to surgical descriptions, placement of external ventricular drainage device and emergency surgeries were more frequently observed in infected patients (*p* < 0.01), and the operative time of infected patients was significantly longer than that of the controls (*p* < 0.05).

**Table 1:** Baseline characteristics of all patients

Characteristics	Infected group (*n* = 131)	Control group (*n* = 150)	*p* value
Male (*n*, %)	64 (49%)	62 (41%)	0.21
Age, years (mean [range])	49 (19—79)	49 (18—74)	0.29
> 65 years (*n*, %)	16 (12%)	15 (10%)	0.56
Medical history (*n*, %)			
Cardiovascular disease	33 (25%)	39 (26%)	0.88
Respiratory disease	6 (5%)	3 (2%)	0.38
Endocrine disease	12 (9%)	10 (7%)	0.44
Central nervous system disease	29 (22%)	34 (23%)	0.92
Digestive system disease	8 (6%)	12 (8%)	0.54
No medical history	43 (33%)	48 (32%)	0.88
History of neurosurgery (*n*, %)	27 (21%)	30 (20%)	0.90
Surgery description (*n*, %)			
Craniotomy for tumor or mass	105 (80%)	109 (73%)	0.14
Craniotomy for cerebrovascular disease	8 (6%)	6 (4%)	0.42
Craniotomy for epilepsy	2 (2%)	3 (2%)	1.00
Craniotomy for others	3 (2%)	5 (3%)	0.87
Transphenoidal or transnasal	13 (10%)	27 (18%)	0.05
GCS score on the 1st day after surgery (median [IQR])	15 (13—15)	15 (14–15)	0.52
Drainage devices before infection (*n*, %)	71 (54%)	67 (45%)	0.11
External ventricular drainage	57 (80%)	39 (58%)	< 0.01*
Lumber cistern drainage	14 (20%)	28 (42%)	0.06
Emergency surgery (*n*, %)	13 (10%)	3 (2%)	< 0.01*
Operative time, hours (median [IQR])	4.7 (3.8—6.1)	4.2 (3.3—5.6)	0.03 *
Glucocorticoids administration (*n*, %)	111 (85%)	134 (89%)	0.25
Antimicrobial prophylaxis (*n*, %)	125 (95%)	148 (99%)	0.20
Empirical antibiotic therapy before sampling (*n*, %)	45 (34%)	62 (41%)	0.23
Vital signs (*n*, %)			
Fever (> 38 ℃)	109 (83%)	–	–
Headache	50 (38%)	–	–
Neck stiffness	32 (24%)	–	–
Altered consciousness or other symptoms	18 (14%)	–	–
Abnormal cerebrospinal parameters	126 (96%)	–	–
Positive cultures at the time of diagnosis	5 (4%)	–	–
Days from surgery to infection (median [IQR])	5 (3—7)	–	–
Patients with positive cultures (*n*, %)	35 (27%)	–	–
CSF cytochemical parameters (median [IQR])			
White blood cell count (cells/mm^3^)	2201 (809—6207)	19 (8—39)	< 0.01*
Protein (mg/dL)	175 (120—359)	54 (39—81)	< 0.01*
Glucose (mmol/L)	2.0 (1.2—2.2)	3.6 (3.1—4.2)	< 0.01*

IQR interquartile range. GCS score Glasgow Coma Scale score. CSF cerebrospinal fluid. * Values that with significant differences

– There was no value in this part

The median time to onset of meningitis and ventriculitis was 5 days (IQR 3—7 days) post-neurosurgery. There were 126 patients who established a diagnosis based on abnormal cerebrospinal parameters combined with corresponding clinical manifestations. The other 5 patients had a normal white blood cell count or glucose level and were confirmed to be infected in light of positive cultures in CSF. The causal pathogens included 3 cases of coagulase-negative staphylococci, 1 case of *Klebsiella pneumoniae* and 1 case of *Stenotrophomonas maltophilia*.

### Isolated organisms

A total of 35 patients were determined to be infected by positive cultures of CSF or blood, containing 28 patients with positive CSF cultures, 1 patient with positive blood culture and 6 patients with both positive cultures. As summarized in Table 2, 34 patients contributed to 41 CSF culture-positive episodes, and 7 patients contributed to 7 blood culture-positive episodes. Overall, Gram-negative bacteria were responsible for most infections, which were isolated from 21 (51%) patients, slightly above 20 (49%) of Gram-positive bacteria. Mixed infections occurred in 7 (20%) of the culture-positive patients. Repeated positive cultures were documented in 16 patients, and each of the other 19 patients showed a positive culture only from one single sample, including 7 episodes of coagulase-negative staphylococci, 4 episodes of *Enterococcus spp.*, 3 episodes of *Klebsiella pneumoniae*, 3 episodes of *Acinetobacter baumannii*, 1 episode of *Staphylococcus aureus* and 1 episode of *Raoultella ornithinolytica*. The most frequently isolated organism was coagulase-negative staphylococci, followed by *Klebsiella pneumoniae* and *Acinetobacter baumannii*. Rare pathogens recorded included *Raoultella ornithinolytica*, *Serratia marcescens*, *Citrobacter*, *Providencia stuartii* and *Alcaligenes faecalis*.

**Table 2:** Organisms isolated from blood and cerebrospinal fluid

Isolated organisms	CSF cultures	Blood cultures
Coagulase-negative staphylococci	13	2
*Staphylococcus aureus*	1	0
*Enterobacter spp.*	2	1
*Enterococcus spp.*	6	2
*Klebsiella pneumoniae*	8	1
*Acinetobacter spp.*	6	1
*Stenotrophomonas maltophilia*	3	0
*Alcaligenes faecalis*	1	0
*Raoultella ornithinolytica*	1	0
Total episodes	41	7

CSF cerebrospinal fluid

### Diagnostic accuracy of CSF HBP

**Fig. 1:**
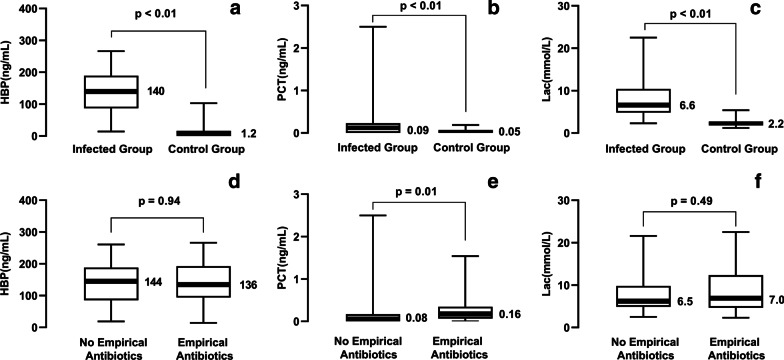
Levels of heparin-binding protein, procalcitonin and lactate in cerebrospinal fluid. The median levels were labeled in the figures. The values were described as medians (interquartile ranges [IQR]) and listed below: **a** Heparin-binding protein (HBP) were 140 ng/mL (IQR 87—189 ng/mL) in the infected patients versus 1.2 ng/mL (IQR 0.7—3.5 ng/mL) in the control patients. **b** Procalcitonin (PCT) were 0.09 ng/mL (IQR 0.00—0.23 ng/mL) in the infected patients versus 0.05 ng/mL (IQR 0.00—0.07 ng/mL) in the control patients. **c** Lactate (Lac) were 6.6 mmol/L (IQR 4.8—10.4 mmol/L) in the infected patients versus 2.2 mmol/L (IQR 1.8—2.7 mmol/L) in the control patients. **d** HBP were 136 ng/mL (IQR 94—193 ng/mL) in the group with empirical antibiotics versus 144 ng/mL (IQR 86—188 ng/mL) in the group without empirical antibiotics. **e** PCT were 0.16 ng/mL (IQR 0.07—0.35 ng/mL) in the group with empirical antibiotics versus 0.08 ng/mL (IQR 0.00—0.17 ng/mL) in the group without empirical antibiotics.** f** Lactate were 7.0 mmol/L (IQR 4.7—12.4 mmol/L) in the group with empirical antibiotics versus 6.5 mmol/L (IQR 4.9—9.8 mmol/L) in the group without empirical antibiotics

All three parameters in CSF presented universal increases in the infected group (*p* < 0.01) (Fig. 1a–c). CSF HBP increased notably in patients with postoperative meningitis or ventriculitis (median 140 ng/mL [IQR 87—189 ng/mL]) compared to patients in whom central nervous system infection was finally ruled out (median 1.2 ng/mL (IQR 0.7—3.5 ng/mL). (Fig. 1a).

**Fig. 2:**
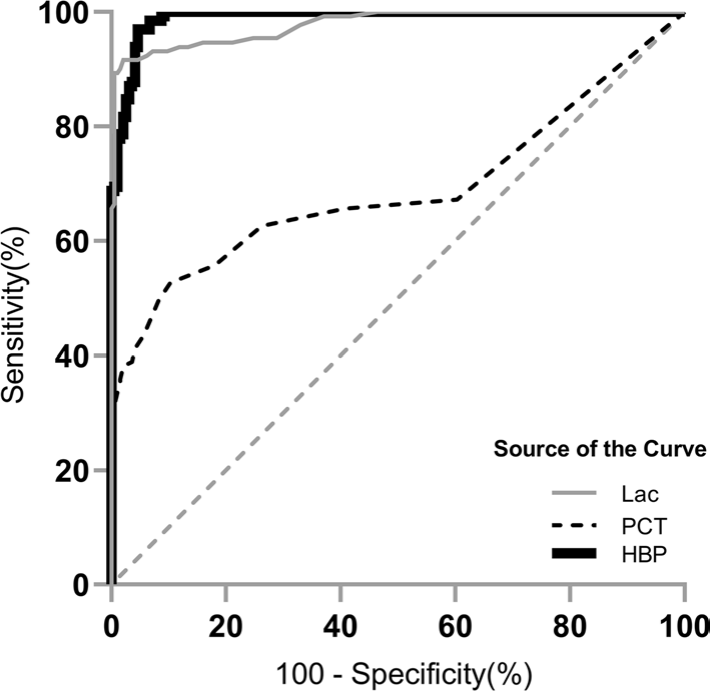
ROC curves of cerebrospinal fluid biomarkers for diagnosing nosocomial meningitis and ventriculitis. The area under the receiver operating characteristic curve (AUC) was 0.99 (95% CI 0.98—1.00) for heparin-binding protein (HBP), 0.98 (95% CI 0.96—0.99) for lactate (Lac) and 0.69 (95% CI 0.62—0.75) for procalcitonin (PCT)

The receiver operating characteristic curve (ROC curve) for the discrimination of nosocomial meningitis and ventriculitis indicated that CSF HBP achieved the largest AUC of 0.99 (95% confidence interval [CI], 0.98—1.00), which was higher than the other two biomarkers: 0.98 (95% CI 0.96—0.99) for CSF lactate and 0.69 (95% CI 0.62—0.75) for PCT (Fig. 2).

A cutoff level of CSF HBP at 23 ng/mL identified postoperative meningitis and ventriculitis with a sensitivity of 97%, a specificity of 95%, a positive predictive value of 93% and a negative predictive value of 98%. With a cutoff level at 103 ng/mL, CSF HBP presented a specificity of 100%, but the sensitivity decreased to 69% (Table 3).

**Table 3:** Diagnostic efficiency of cerebrospinal fluid biomarkers

Parameters	All patients			EVD-related infections		
	HBP (ng/mL)	Procalcitonin (ng/mL)	Lactate (mmol/L)	HBP (ng/mL)	Procalcitonin (ng/mL)	Lactate (mmol/L)
Cutoff level	> 23	> 0.09	> 3.9	> 21	> 0.09	> 3.9
Sensitivity (%)	97	53	92	100	54	88
Specificity (%)	95	90	98	96	89	100
Positive predictive value (%)	93	78	97	100	54	88
Negative predictive value (%)	98	74	94	95	87	100

CSF cerebrospinal fluid. HBP heparin-binding protein. EVD-related infections external ventricular drainage-related infections

### The value of CSF HBP in identifying bacterial meningitis and ventriculitis

**Fig. 3:**
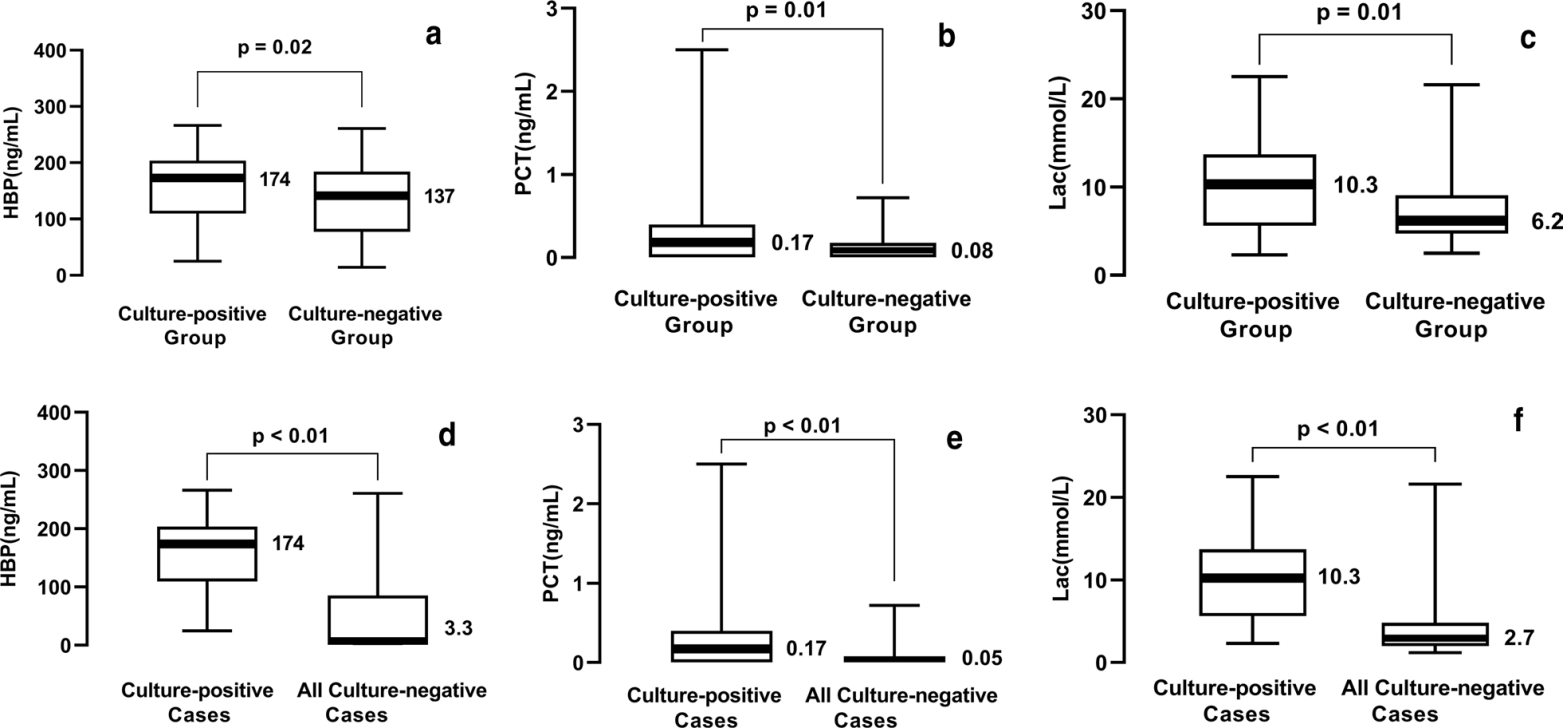
Levels of heparin-binding protein, procalcitonin and lactate in cerebrospinal fluid of culture-positive patients. HBP were 174 ng/mL (IQR 110—204 ng/mL) in the culture-positive group versus 137 ng/mL (IQR 77—184 ng/mL) in the culture-negative group **(a)** and 3.3 ng/mL (IQR 0.9—85.2 ng/mL) in all culture-negative cases **(d)**. CSF PCT were 0.17 ng/mL (IQR 0.00—0.40 ng/mL) in the culture-positive group versus 0.08 ng/mL (IQR 0.00—0.18 ng/mL) in the culture-negative group **(b)** and 0.05 ng/mL (IQR 0.00—0.08 ng/mL) in all culture-negative patients **(e)**. Lactate were 10.3 mmol/L (IQR 5.6—13.7 mmol/L) in the culture-positive group versus 6.2 mmol/L (IQR 4.7—9.1 mmol/L) in the culture-negative group **(c)** and 2.7 mmol/L (IQR 2.0—4.8 mmol/L) in all culture-negative patients **(f)**

The infected group (*n* = 131) was further divided into the culture-positive group (*n* = 35) and the culture-negative group (*n* = 96) based on bacterial findings in blood or CSF (Fig. 3a–c). The comparison was also performed on the culture-positive patients (*n* = 35) and all culture-negative cases (*n* = 246) (Fig. 3d–f). HBP levels in all culture-positive patients exceeded 23 ng/mL, including the patients who had a negative white blood cell count or glucose level and were confirmed infection by positive culture results, 28 (80%) cases among them had HBP levels above 103 ng/mL. Concentrations of all three parameters in CSF were significantly higher in patients with positive cultures. CSF HBP were 174 ng/mL (IQR 110—204 ng/mL) in the culture-positive group versus 137 ng/mL (IQR 77—184 ng/mL) in the culture-negative group (*p* < 0.05) and 3.3 ng/mL (IQR 0.9—85.2 ng/mL) in all culture-negative cases (*p* < 0.05).

CSF PCT presented a low sensitivity in the diagnosis of nosocomial meningitis and ventriculitis. Forty-three (33%) patients had PCT levels of less than 0.05 ng/mL, and 62 (47%) patients had PCT levels of less than 0.09 ng/mL (the cutoff level) among the 131 infected patients. Even among the 35 culture-positive patients, 9 (26%) had CSF PCT levels lower than 0.05 ng/mL.

### Diagnostic accuracy of CSF HBP in patients who received empiric antibiotics

Empiric antimicrobial therapy prior to sampling was documented in 45 patients (34%), including 20 patients who were treated for more than 48 h. There was no statistical significance in the concentrations of CSF HBP and lactate between the patients who received empiric antibacterial therapy (*n* = 45) and those who did not (*n* = 86) (*p* > 0.05, Fig. 1d, f), while the levels of CSF PCT in the group with empiric antibiotics were significantly higher than those in the group without (Fig. 1e). The characteristics of patients with and without empiric antibiotics were compared to look for possible causative factors for this discrepancy. There were 19 (42%) critically ill patients and 19 (42%) culture-positive patients in the group with empiric antibiotic use versus 19 (22%) critically ill cases and 16 (19%) culture-positive cases among patients without antibiotic pretreatment. The median time to onset of infection was 7 days (IQR 4—10 days) post-neurosurgery in patients who were pretreated versus 4 days (IQR 2—6 days) post-neurosurgery in those who were not pretreated. The levels of the biomarkers were further compared between critically ill patients with and without empiric antibiotics, noncritically ill patients with and without empiric antibiotic use respectively. Concentrations of PCT showed no statistical significance between the two pairs of subgroups, *p* = 0.15 and *p* = 0.17 respectively.

Here, we observed that 43 (96%) cases had HBP concentrations above 23 ng/mL among the 45 patients who were treated with empiric anti-infective therapy, whereas the PCT levels were below 0.05 ng/mL in 9 (20%) patients and less than 0.09 ng/mL in 18 patients (40%).

### Diagnostic accuracy of CSF HBP in EVD-related infections

As shown in Table 1, 96 patients with EVD devices were enrolled, 57 (44% of all the infected patients and 59% of patients with EVD devices) were diagnosed with meningitis or ventriculitis, and in 39 (26% of the total control patients and 41% of patients with EVD devices), central nervous system infection was finally ruled out. The median levels of the markers were: 154 ng/mL (IQR 110—192 ng/mL) for HBP in patients with EVD-related infections versus 1.6 ng/mL (IQR 0.7—3.9 ng/mL) in those without (*p* < 0.01), 0.09 ng/mL (IQR 0.00—0.27 ng/mL) for PCT in patients with EVD-related infections versus 0.05 ng/mL (IQR 0.00—0.07 ng/mL) in those without (*p* < 0.01) and 8.2 mmol/L (IQR 4.7—12.5 mmol/L) for lactate in patients with EVD-related infections versus 2.2 mmol/L (IQR 1.8—2.8 mmol/L) in those without (*p* < 0.01). The AUCs for the diagnosis of EVD-related infections were 0.99 (95% CI 0.98—1.00) for HBP, 0.97 (95% CI 0.95—1.00) for lactate and 0.72 (95% CI 0.62—0.82) for PCT. HBP achieved a sensitivity of 100% and a specificity of 96% with a cutoff value at 21 ng/mL. (Table 3).

### Correlation between CSF biomarkers

After adjustment by using Spearman's partial correlation, there was still a strong association between HBP and WBC (Spearman's rho = 0.57, *p* < 0.01), as well as HBP and polymorphonuclear cell (PMN) (Spearman's rho = 0.68, *p* < 0.01). But the correlation between WBC and lactate (Spearman's rho = 0.38, *p* < 0.01), as well as HBP and lactate (Spearman's rho = 0.34, *p* < 0.01), was relatively weak. (Table 4).

**Table 4:** Spearman's correlation coefficients for cerebrospinal fluid biomarkers

Biomarkers	Spearman's rank correlation coefficients	*p* value		Spearman's partial correlation coefficients	*p* value
HBP—PMN	0.86	*p* < 0.01		0.68	*p* < 0.01
HBP—WBC	0.81	*p* < 0.01		0.57	*p* < 0.01
WBC—lactate	0.75	*p* < 0.01		0.38	*p* < 0.01
HBP—lactate	0.74	*p* < 0.01		0.34	*p* < 0.01

HBP heparin-binding protein. WBC white blood cell. PMN polymorphonuclear cell

## Discussion

In the present study, we investigated the diagnostic accuracy of cerebrospinal fluid heparin-binding protein in patients with nosocomial meningitis or ventriculitis compared to procalcitonin and lactate and performed further subgroup analysis based on whether these patients had positive culture findings and whether empiric antibiotics were used. Our study highlights the discriminatory value of CSF HBP and contributes to solving the diagnostic difficulties caused by empiric antibacterial treatment for the diagnosis of nosocomial meningitis and ventriculitis.

The protein demonstrated superior diagnostic capability in distinguishing patients with postoperative meningitis or ventriculitis as shown by the largest AUC in comparison with CSF PCT and lactate. Similar results were observed in the diagnosis of EVD-related infections. Published studies revealed that CSF lactate had a higher diagnostic accuracy with a cutoff level at 4 mmol/L compared to conventional CSF markers, including glucose, protein and white blood cell count [23, 24]. However, its diagnostic value may be limited when CSF samples are mixed with blood [25], which is common in patients following neurosurgery, and normal levels of lactate may not rule out all infected patients [26]. Our study justified that CSF HBP better recognized postoperative meningitis and ventriculitis than lactate, but with modest differences and overlapping confidence intervals. HBP presented a higher sensitivity and negative predictive value but a lower specificity and positive predictive value than lactate, while lactate showed the opposite results. This indicates that the measurement of CSF HBP may be more helpful in identifying infection, with a low risk of missed diagnosis, and elevated lactate values are more indicative of an existing infection, with a low risk of misdiagnosis. Detecting the two markers simultaneously may be more helpful in this setting.

Contrary to previous studies [19, 20], our research showed significant positive correlation between HBP and CSF WBC and an even more notable correlation between HBP and CSF PMN. While the correlation between HBP and lactate was weak. Previous studies did not consider the mutual influence between the markers, which likely explains these differences. Compared with WBC and lactate, WBC and HBP presented a stronger positive correlation after adjusting. This revealed a potential risk of overestimating the diagnostic performance of HBP, since HBP is generated from activated neutrophils and we adopted leukocytosis as one of the diagnostic criteria.

The findings that CSF HBP increased in all culture-positive cases indicate that HBP could have a large clinical use in early identification of patients with a bacterial infection, especially in those with otherwise negative cytochemistry. Clinicians should be cautious when neurosurgical patients have a normal CSF leukocyte count and CSF glucose level but an elevated HBP level in CSF, they should be assumed to have bacterial meningitis until a negative CSF culture is obtained. Our results are in agreement with previous clinical investigations on community-acquired meningitis [19, 20]. Stored in mature neutrophils [14], HBP can be mobilized and released from neutrophils immediately under the stimulation of bacterial pathogens [15, 27]. Adam Linder and coworkers found that the levels of HBP most likely reflected the cell count of functional neutrophils, other than the absolute number of white blood cells [19]. This may partly explain the striking elevation of CSF HBP in patients with positive bacteriological results and a negative white blood cell count.

It should also be mentioned that there is a considerable overlap in HBP concentrations between the culture-positive and culture-negative cases in the infected group, which indicates that HBP may not be able to fully discriminate bacterial meningitis from culture-negative meningitis, and there may be subjects with chemical meningitis in the culture-negative group. The problem is hard to address since the CDC/NHSN definition allows for diagnosis of culture-negative meningitis.

As the sensitivity of culture and Gram staining is low, patients without microbiological confirmation are clinically more common. Most of the infected patients were diagnosed based on clinical symptoms and CSF cytochemistry abnormalities at enrollment in our study, which is more in line with clinical practice, but this may not help to identify causal pathogens. In fact, infections were verified largely depending on subsequent bacteriological results and responsiveness to empiric antibacterial therapy. We excluded from the study patients who met only one or two of the following criteria: decreased CSF glucose, pleocytosis and increased protein. However, these patients may also have symptoms such as low fever or headache, and clinicians tend to initiate antibacterial treatment. It was difficult to define whether such patients were infected or not and were thereby excluded. Therefore, a risk of misclassification may exist.

The IDSA guideline defines that positive cultures with normal CSF cytochemical parameters and no suspicious clinical manifestations as contamination (or colonization), that positive cultures with leukocytosis and clinical symptoms as infection [22]. There was a possibility that the bacteria grew were not the causal pathogens, especially in patients who had only one positive culture. But the effectiveness of targeted antibacterial treatment against these pathogens may help to identify whether the isolated bacteria were responsible. In our study, all culture-positive patients presented clinical manifestations of suspected infections, and most of them also had CSF abnormalities except for 5 patients who had positive cultures and a normal CSF leukocyte count or glucose level. Three of the 5 culture-positive patients had a normal CSF cell count and all isolated bacteria were coagulase-negative staphylococci. Thus, a possibility of erroneously judging contamination (or colonization) as infection may exist in these 3 patients.

CSF PCT presented limited identifiable value and poor sensitivity for the diagnosis of nosocomial meningitis and ventriculitis compared with the other parameters we tested. Our findings are comparable with figures evaluated in previous observations [28, 29]. Wen Jiang and colleagues found that CSF PCT aided in the discrimination of Gram-negative bacterial meningitis [30]. However, our findings are not contradictory to theirs. In fact, the median levels of CSF PCT in patients with nosocomial meningitis or ventriculitis did increase with meaningful statistical difference, and its concentrations in patients with positive cultures were also significantly higher than those without, but not all infected patients contributed to the elevation of PCT values. PCT concentrations in a substantial portion of patients with meningitis or ventriculitis were recorded as negative in our study, and even a quarter of culture-positive patients had PCT levels below the lower limit of detection. Therefore, a significant increase in CSF PCT may indicate infections, but normal PCT levels cannot exclude all infected patients, suggesting that PCT may be more of a marker that implies an already existing infection rather than predicting infections in the specific setting of neurosurgery.

Suspicion of central nervous system infection following neurosurgery usually warrants empiric antibiotic prescription. However, this may blunt the changes of CSF markers, reduce the sensitivity of CSF microbiological testing and mask the typical clinical manifestations [31, 32]. Here, we observed that most of the infected patients who were already on antibiotics prior to sampling had elevated HBP concentrations at the time of diagnosis (exceeding 23 ng/mL), including those who were treated for more than 48 h, suggesting that HBP retains a high diagnostic ability in patients who received antibiotic pretreatment. How antibiotic pretreatment modifies these parameters in CSF requires further study.

Unexpectedly, our study suggested that patients who received empiric antimicrobial chemotherapy had significantly higher CSF PCT levels than those who did not. Comparison of the basic characteristics of the two groups showed that the portion of critically ill cases and culture-positive patients was greater and the median time to onset of infection was longer in the group given empiric antibiotics. Given these findings, we speculate that the reasons may be as follows. Mostly synthesized by the parafollicular C cells of the thyroid gland and derived largely from plasma penetration, CSF PCT may increase slowly and reach peak values at a relatively late stage of meningitis and ventriculitis. At the same time, patients who were given antibiotic intervention in advance were less likely to present typical clinical manifestations in the early period. Thus, the time from surgery to establish the diagnosis of meningitis and ventriculitis will be prolonged. The overlap of these two effects was probably the major reason that patients given empiric anti-infective therapy had a higher PCT value. Moreover, the discrepancy in the proportion of critically ill patients between the two subgroups may also be responsible. Given the catastrophic consequences that delayed antibiotics may incur, critically ill patients are more likely to initiate antibacterial treatment when suspected to have central nervous system infections, despite insufficient evidence. The PCT levels of critically ill patients may be higher than those of mild patients [33]. This made patients with empiric antibiotic pretreatment appear to have a higher PCT level. Studies on CSF PCT have mostly been of low-quality until recently, and our hypothesis needs to be confirmed by further research.

Further subgroup analysis revealed that regardless of whether critically ill or not, all three biomarkers were not significantly different between patients who received or did not receive empiric antibiotherapy. These results correspond to the findings of previous prospective studies [19, 28, 31]. However, CSF PCT is of questionable value for its poor sensitivity in diagnosing nosocomial meningitis and ventriculitis in patients with empiric antibiotic use. These findings highlight that the ability of CSF HBP to diagnose postoperative meningitis and ventriculitis will not decrease in the context of antimicrobial chemotherapy preceding diagnosis, and it may be a useful marker that contributes to resolving the diagnostic dilemmas in these patients.

The novelty of this research was that we assessed the efficacy of CSF HBP in diagnosing nosocomial meningitis and ventriculitis while previous studies were mainly designed for community-acquired meningitis [18–20]. Moreover, our research enrolled patients given anti-infective therapy prior to diagnosis, which is more consistent with the actual clinical situation, while previous studies mostly excluded these patients. Additionally, our study enrolled a larger sample size.

The limitations of the present study may be as follows. Patients admitted due to tumor surgery predominate in our subjects, which can increase patient homogeneity but may lead to selection bias as well, and we should be cautious when extrapolating our findings to patients with diseases other than tumors. In addition, definitions of postoperative meningitis adopted in different studies differ significantly, which makes the range of patient populations recruited vary. Our research used an internationally recognized definition to minimize the difference, which may have made strict choices for enrolled patients inadvertently.

## Conclusions

Cerebrospinal fluid heparin-binding protein is a reliable auxiliary diagnostic marker outperforming lactate and procalcitonin in the identification of nosocomial meningitis and ventriculitis, and it contributes to solving the diagnostic difficulties caused by empirical antibacterial treatment.

## Data Availability

All data generated or analyzed during this study are included in this published article. The datasets generated or analyzed during the current study are available from the corresponding author on reasonable request.

## Abbreviations

HBP: Heparin-binding protein
PCT: Procalcitonin
Lac: Lactate
CSF: Cerebrospinal fluid
IQR: Interquartile range
CI: Confidence interval
GCS score: Glasgow Coma Scale score
ROC curve: Receiver operating characteristic curve
AUC: The area under receiver operating characteristic curve
PMN: Polymorphonuclear cell
WBC: White blood cell
EVD-related infections: External ventricular drainage-related infections
IDSA: Infectious Diseases Society of America
ICU: The intensive care unit
